# Are there any differences in clinical and biochemical variables between bipolar patients with or without lifetime psychotic symptoms?

**DOI:** 10.1192/j.eurpsy.2023.1074

**Published:** 2023-07-19

**Authors:** C. M. Esposito, A. Ceresa, M. Di Paolo, T. Surace, L. Moioli, F. Legnani, G. Nosari, V. Ciappolino, A. M. Auxilia, M. Cappellazzi, I. Tagliabue, L. Cirella, E. Capuzzi, A. Caldiroli, A. Dekanalis, M. Clerici, M. Buoli

**Affiliations:** 1 Department of Pathophysiology and Transplantation, University of Milan, Milano; 2 Psychiatric Department, Azienda Socio-Sanitaria Territoriale Monza, Monza; 3 University of Milan; 4 Department of Neurosciences and Mental Health, Fondazione IRCCS Ca’ Granda Ospedale Maggiore Policlinico, Milano; 5 Department of Medicine and Surgery, University of Milano Bicocca, Monza; 6 Department of Medicine and Surgery, University of Milano Bicocca; 7 Healthcare Professionals Department, Fondazione IRCCS Ca’ Granda Ospedale Maggiore Policlinico, Milano, Italy

## Abstract

**Introduction:**

Bipolar Disorder (BD) is a frequent psychiatric disorder, which can be associated with high disability. Psychotic symptoms occur in more than half of bipolar patients and are associated with an unfavorable course of the disorder (Chakrabarti *et al.* World J Psychiatry 2022; 12(9) 1204-1232).

**Objectives:**

The aim of this study is therefore to identify clinical and biological markers able to discriminate between BD patients with (BD-PS) and without lifetime psychotic symptoms (BD-NPS) to facilitate early diagnosis and to implement a target clinical management of these patients.

**Methods:**

We recruited 665 patients consecutively hospitalized for BD at Fondazione IRCCS Policlinico (Milan) and at San Gerardo Hospital (Monza). Data were obtained through a screening of the clinical charts and blood analyses conducted during the hospitalization. Patients were assessed by psychometric scales. The two groups (BD-PS and BD-NPS) were compared by t tests for quantitative variables and χ^2^ tests for qualitative ones. Variables that resulted to be significant in univariate analyses were inserted in binary logistic models with the presence of psychotic symptoms as dependent variable.

**Results:**

Among the total sample, 64.5% of patients were affected by BD-PS while 35.5% by BD-NPS. The final binary logistic regression model showed that, compared to patients with BD-NPS, those with BD-PS had a longer duration of hospitalization (p=0.007) and were more frequently hospitalized for a manic episode (p=0.001). In addition, subjects with BD-PS had a lower score on the current Global Assessment of Functioning (GAF) (t = 3.157; p = 0.002) and were more frequently males (χ² = 4.061; p = 0.044; OR = 1.399). With regard to biological variables, patients with BD-PS, compared to the counterpart, had a higher Neutrophile to Lymphocyte Ratio (NLR) (t = 2.776; p = 0.006), lower levels of Gamma-Glutamyl Transferase (γGT) (t = 2.249; p = 0.026), higher total bilirubin (t = 2.348; p = 0.019) and creatine phosphokinase (CPK) (t=2.807; p = 0.005), lower total cholesterol (t = 2.369; p = 0.018) and triglycerides (t = 2.554; p = 0.013).

**Conclusions:**

Our data appear to be in line with the literature, especially with respect to the occurrence of psychotic symptoms mainly in manic episodes and their association with greater clinical severity, longer hospitalization and worse outcome (Altamura *et al*. Aust N Z J Psychiatry 2019; 53(8) 772-781). From a biological point of view, it seems important to emphasize that patients with lifetime psychotic symptoms presented a higher NLR, revealing more prominent low-grade inflammation in these patients than the counterpart. These data confirm the possibility of using NLR as biomarker of severity in bipolar patients, as proposed previously by other authors (Kulacaoglu *et al.* Nord J Psychiatry 2022). Future multi-center study have to confirm the results of the present study.

**Disclosure of Interest:**

None Declared

